# Supplementary material for: Tactile localization of the breast, areola, and nipple
Source: eLife. 2026 Jun 11;14:RP105946. doi: 10.7554/eLife.105946 (PMC13258733; doi:10.7554/eLife.105946)
Supplement: Peer review history

# Editor report: eLife Assessment

**DOI:** 10.7554/eLife.105946.4.sa0

**Authors:** Tamar R Makin

**Affiliations:** University of Cambridge United Kingdom

**Keywords:** Convincing, Important

This study thoroughly assesses tactile acuity on women's breasts, for which no dependable data currently exists. The study provides two **important** contributions, by **convincingly** showing that tactile acuity on the breast is poor in comparison to other body parts, and that acuity is worst in larger breasts, indicating that the number of tactile sensors is fixed. This study will be of interest to the broader community of touch, as well as those interested in breast reconstruction and sexual function.

# Referee report: Reviewer #1 (Public review)

**DOI:** 10.7554/eLife.105946.4.sa1

[Editors' note: this version has been assessed by the Senior Editor without further input from the original reviewers. The authors have moderated their claims and discussed the limitations of their experimental design more transparently. The previous reviews are included for reference.]

Comments on previous version:

The authors investigated tactile spatial perception on the breast using discrimination, categorization, and direct localization tasks. They reach four main conclusions:

(1) The breast has poor tactile spatial resolution.

This conclusion is based on comparing just noticeable differences, a marker of tactile spatial resolution, across four body regions, two on the breast. The data compellingly support the conclusion; the study outshines other studies on tactile spatial resolution that tend to use problematic measures of tactile resolution, such as two-point-discrimination thresholds. The result will interest researchers in the field and possibly in other fields due to the intriguing tension between the finding and the sexually arousing function of touching the breast.

The manuscript incorrectly describes the result as poor spatial acuity. Acuity measures the average absolute error, and acuity is good when response biases are absent. Precision relates to the error variance. It is common to see high precision with low acuity or vice versa. Just noticeable differences assess precision or spatial resolution, while points of subjective equality evaluate acuity or bias. Similar confusions between these terms appear throughout the manuscript.

A paragraph within the next section seems to follow up on this insight by examining the across-participant consistency of the differences in tactile spatial resolution between body parts. To this aim, pairwise rank correlations between body sites are conducted. This analysis raises red flags from a statistical point of view. (1) An ANOVA and its follow-up tests assume no variation in the size of the tested effect but varying base values across participants. Thus, if significant differences between conditions are confirmed by the original statistical analysis, most participants will have better spatial resolution in one condition than the other condition, and the difference between body sites will be similar across participants. (2) Correlations are power-hungry, and non-parametric tests are power-hungry. Thus, the number of participants needed for a reliable rank correlation analysis far exceeds that of the study. In sum, a correlation should emerge between body sites associated with significantly different tactile JNDs; however, these correlations might only be significant for body sites with pronounced differences due to the sample size.

(2) Larger breasts are associated with lower tactile spatial resolution

This conclusion is based on a strong correlation between participants' JNDs and the size of their breasts. The depicted correlation convincingly supports the conclusion. The sample size is below that recommended for correlations based on power analyses, but simulations show that spurious correlations of the reported size are extremely unlikely at N=18. Moreover, visual inspection rules out that outliers drive these correlations. Thus, they are convincing. This result is of interest to the field, as it aligns with the hypothesis that nerve fibers are more sparsely distributed across larger body parts.

(3) The nipple is a unit

The data do not support this conclusion. The conclusion that the nipple is perceived as a unit is based on poor tactile localization performance for touches on the nipple compared to the areola. The problem is that the localization task is a quadrant identification task with the center being at the nipple. Quadrants for the areola could be significantly larger due to the relative size of the areola and the nipple; the results section seems to suggest this was accounted for when placing the tactile stimuli within the quadrants, but the methods section suggests otherwise. Additionally, the areola has an advantage because of its distance from the nipple, which leads to larger Euclidean distances between the centers of the quadrants than for the nipple. Thus, participants should do better for the areola than for the nipple even if both sites have the same tactile resolution.

To justify the conclusion that the nipple is a unit, additional data would be required. (1) One could compare psychometric curves with the nipple as the center and psychometric curves with a nearby point on the areola as the center. (2) Performance in the quadrant task could be compared for the nipple and an equally sized portion of the areola and tactile locations that have the same distance to the border between quadrants in skin coordinates. (3) Tactile resolution could be directly measured for both body sites using a tactile orientation task with either a two-dot probe or a haptic grating.

Categorization accuracy in each area was tested against chance using a Monte Carlo test, which is fine, though the calculation of the test statistic, Z, should be reported in the Methods section, as there are several options. Localization accuracies are then compared between areas using a paired t-test. It is a bit confusing that once a distribution-approximating test is used, and once a test that assumes Gaussian distributions when the data is Bernoulli/Binomial distributed. Sampling-based and t-tests are very robust, so these surprising choices should have hardly any effect on the results.

A correlation based on N=4 participants is dangerously underpowered. A quick simulation shows that correlation coefficients of randomly sampled numbers are uniformly distributed at such a low sample size. This likely spurious correlation is not analyzed, but quite prominently featured in a figure and discussed in the text, which is worrisome.

(4) Localization of tactile events on the breast is biased towards the nipple

The conclusion that tactile percepts are drawn toward the nipple is based on localization biases for tactile stimuli on the breast compared to the back. Unfortunately, the way participants reported the tactile locations introduces a major confound. Participants indicated the perceived locations of the tactile stimulus on 3D models of these body parts. The nipple is a highly distinctive and cognitively represented landmark, far more so than the scapula, making it very likely that responses were biased toward the nipple regardless of the actual percepts. One imperfect but better alternative would have been to ask participants to identify locations on a neutral grey patch and help them relate this patch to their skin by repeatedly tracing its outline on the skin.

Participants also saw their localization responses for the previously touched locations. This is unlikely to induce bias towards the nipple, but it renders any estimate of the size and variance of the errors unreliable. Participants will always make sure that the marked locations are sufficiently distant from each other.

The statistical analysis is again a homebrew solution and hard to follow. It remains unclear why standard and straightforward measures of bias, such as regressing reported against actual locations, were not used.

Null-hypothesis significance testing only lets scientists either reject the null hypothesis or not. The latter does NOT mean the Null hypothesis is true, i.e., it can never be concluded that there is no effect. This rule applies to every NHST test. However, it raises particular concerns with distribution tests. The only conclusion possible is that the data are unlikely from a population with the tested distribution; these tests do not provide insight into the actual distribution of the data, regardless of whether the result is significant or not.

# Referee report: Reviewer #2 (Public review)

**DOI:** 10.7554/eLife.105946.4.sa2

Summary:

The authors tested tactile acuity on the breast of females using several tasks.

Results:

Tactile acuity, assessed by just-noticeable differences in judging whether a touch was above or below a comparison stimulus, was lower on both the lateral and medial breast than on the hand and back. Acuity also scaled inversely with breast size, echoing earlier findings that larger hands exhibit lower acuity, presumably because a similar number of tactile receptors must be distributed over larger or smaller body surfaces. Observing this principle in the breast as on the hand strengthens the view that fixed innervation is a general organizing principle of the tactile system. Both methodology and analysis appear sound.

Most participants were unable to localize touch to a specific quadrant of the nipple, suggesting it is perceived as a single tactile unit. However, the study does not address whether touches to the nipple and areola are confused; conceptualizing the nipple as a perceptual (landmark) unit would suggest that such confusion should not take place. Aside from this limitation, the methodology and analysis appear sound.

Absolute touch localization, assessed by asking participants to indicate locations on a 3D rendering of their own torso, revealed a bias toward the nipple. The authors interpret this as evidence that the nipple serves as a landmark attracting perceived touch. However, as reviewers noted during review, alternative explanations cannot be fully ruled out: because the stimulus array was centered on the nipple, the observed bias may stem from stimulus distribution rather than landmark status. Aside from this caveat, the methodology and analysis appear sound.

Overall assessment:

The study offers a welcome exception to the prevailing bias in tactile research that limits investigation to the hand and arm. Its support for the fixed innervation hypothesis and its suggestion that the nipple may serve as a potential landmark-though requiring further scrutiny-illustrate the value of extending research to other body regions. By employing multiple tasks, the authors address several key aspects of tactile perception and create links to earlier findings.

# Author comment: Author response

**DOI:** 10.7554/eLife.105946.4.sa3

**Authors:** Katie H Long, Emily E Fitzgerald, Ev I Berger-Wolf, Amani Fawaz, Stacy T Lindau, Sliman J Bensmaia, Charles M Greenspon

**Affiliations:** University of Chicago Chicago United States; University of Chicago Chicago United States; University of Chicago Chicago United States; University of Chicago Chicago United States; University of Chicago Chicago United States; University of Chicago Chicago United States; University of Chicago Chicago United States

The following is the authors’ response to the previous reviews

**Public Reviews:**

**Reviewer #1 (Public review):**

The manuscript incorrectly describes the result as poor spatial acuity. Acuity measures the average absolute error, and acuity is good when response biases are absent. Precision relates to the error variance. It is common to see high precision with low acuity or vice versa. Just noticeable differences assess precision or spatial resolution, while points of subjective equality evaluate acuity or bias. Similar confusions between these terms appear throughout the manuscript.

While I do not agree with the reviewer's usage of the word “acuity” and a cursory Google search does not agree with the provided definition, I have replaced acuity with precision as appropriate to improve clarity.

A paragraph within the next section seems to follow up on this insight by examining the across-participant consistency of the differences in tactile spatial resolution between body parts. To this aim, pairwise rank correlations between body sites are conducted. This analysis raises red flags from a statistical point of view. (1) An ANOVA and its follow-up tests assume no variation in the size of the tested effect but varying base values across participants. Thus, if significant differences between conditions are confirmed by the original statistical analysis, most participants will have better spatial resolution in one condition than the other condition, and the difference between body sites will be similar across participants. (2) Correlations are power-hungry, and non-parametric tests are power-hungry. Thus, the number of participants needed for a reliable rank correlation analysis far exceeds that of the study. In sum, a correlation should emerge between body sites associated with significantly different tactile JNDs; however, these correlations might only be significant for body sites with pronounced differences due to the sample size.

We have entirely removed this result from both the text and supplement.

The data do not support this conclusion. The conclusion that the nipple is perceived as a unit is based on poor tactile localization performance for touches on the nipple compared to the areola. The problem is that the localization task is a quadrant identification task with the center being at the nipple. Quadrants for the areola could be significantly larger due to the relative size of the areola and the nipple; the results section seems to suggest this was accounted for when placing the tactile stimuli within the quadrants, but the methods section suggests otherwise. Additionally, the areola has an advantage because of its distance from the nipple, which leads to larger Euclidean distances between the centers of the quadrants than for the nipple. Thus, participants should do better for the areola than for the nipple even if both sites have the same tactile resolution.

We agree with this interpretation and have updated the language throughout.

Categorization accuracy in each area was tested against chance using a Monte Carlo test, which is fine, though the calculation of the test statistic, Z, should be reported in the Methods section, as there are several options. Localization accuracies are then compared between areas using a paired t-test. It is a bit confusing that once a distribution-approximating test is used, and once a test that assumes Gaussian distributions when the data is Bernoulli/Binomial distributed. Sampling-based and t-tests are very robust, so these surprising choices should have hardly any effect on the results.

Excellent point. We have replaced the paired t-test with a signed rank test and added text to the methods to expand upon this.

A correlation based on N=4 participants is dangerously underpowered. A quick simulation shows that correlation coefficients of randomly sampled numbers are uniformly distributed at such a low sample size. This likely spurious correlation is not analyzed, but quite prominently featured in a figure and discussed in the text, which is worrisome.

We have removed this panel to reduce this concern.

The conclusion that tactile percepts are drawn toward the nipple is based on localization biases for tactile stimuli on the breast compared to the back. Unfortunately, the way participants reported the tactile locations introduces a major confound. Participants indicated the perceived locations of the tactile stimulus on 3D models of these body parts. The nipple is a highly distinctive and cognitively represented landmark, far more so than the scapula, making it very likely that responses were biased toward the nipple regardless of the actual percepts. One imperfect but better alternative would have been to ask participants to identify locations on a neutral grey patch and help them relate this patch to their skin by repeatedly tracing its outline on the skin.

While I wholeheartedly agree with the sentiments of the reviewer, in our experience performing these tests across many women we have found that the variability of the morphology of the breast makes it incredibly hard for women to perform this task in the way the reviewer is describing. Consequently, there is likely no perfect version of the task. That said, we have endeavored to acknowledge the limitations of the approach in the discussion.

Participants also saw their localization responses for the previously touched locations. This is unlikely to induce bias towards the nipple, but it renders any estimate of the size and variance of the errors unreliable. Participants will always make sure that the marked locations are sufficiently distant from each other.

I again respectfully disagree with this interpretation. If the participants were to always make sure marked locations were sufficiently distant from each other then the degree of error and bias would be similar between regions given that the visual pattern would be almost identical. As this is not true in the data, I disagree with the premise, though we hope the changes to the discussion acknowledge limitations with the data collection method.

Null-hypothesis significance testing only lets scientists either reject the null hypothesis or not. The latter does NOT mean the Null hypothesis is true, i.e., it can never be concluded that there is no effect. This rule applies to every NHST test. However, it raises particular concerns with distribution tests. The only conclusion possible is that the data are unlikely from a population with the tested distribution; these tests do not provide insight into the actual distribution of the data, regardless of whether the result is significant or not.

Thank you for this comment. We have updated the language to make it explicit that we do not mean to imply failing to deviate from the Null distribution does not mean that they are in fact Null in nature.

**Recommendations for the authors:**

**Reviewer #2 (Recommendations for the authors):**

I am wondering whether the interpretation of "the nipple as a sensory unit" is also supported by localization performance as reported in the analysis around Fig. 3 and supplementary Fig. 2. I cannot really see the error lines in that figure, and cannot tell whether any of the touches were on the nipple proper. Specifically I am wondering whether touch to the nipple is reliably attributed to the nipple, and touch to the areola to the areola, or whether confusion exists between the two. The description of the nipple as a sensory unit implies reliable attribution of touch to the respective area. Also the discussion (lines 309ff) is ambiguous about this.

Thank you for this comment. We have removed language about the nipple being a unit and reframed the text in the discussion. We have also clarified that touches were indeed on the nipple.

typos etc.

lines 68-71 - implied causality is not backed up by evidence and could be the other way around than stated here

line 82 grammar is inconsistent

lines 199-200, "on the nipple" occurs twice

Thank you for catching these. We have addressed the typos and grammar. We have also added a citation to the sentence where this exact hypothesis is stated. We have also relaxed the language to imply it is indeed a hypothesis.
